# Development and validation of a nomogram for predicting stroke risk in rheumatoid arthritis patients

**DOI:** 10.18632/aging.203071

**Published:** 2021-06-03

**Authors:** Fangran Xin, Lingyu Fu, Bowen Yang, Haina Liu, Tingting Wei, Cunlu Zou, Bingqing Bai

**Affiliations:** 1 Department of Clinical Epidemiology and Evidence-Based Medicine, The First Affiliated Hospital, China Medical University, Shenyang, China; 2 Department of Medical Record Management Center, The First Affiliated Hospital, China Medical University, Shenyang, China; 3 Department of Rheumatology, The First Affiliated Hospital, China Medical University, Shenyang, China; 4 Neusoft Research of Intelligent Healthcare Technology, Co. Ltd., Shenyang, China

**Keywords:** rheumatoid arthritis, stroke, lipids, inflammatory markers, development and validation nomogram

## Abstract

We developed and validated a nomogram to predict the risk of stroke in patients with rheumatoid arthritis (RA) in northern China. Out of six machine learning algorithms studied to improve diagnostic and prognostic accuracy of the prediction model, the logistic regression algorithm showed high performance in terms of calibration and decision curve analysis. The nomogram included stratifications of sex, age, systolic blood pressure, C-reactive protein, erythrocyte sedimentation rate, total cholesterol, and low-density lipoprotein cholesterol along with the history of traditional risk factors such as hypertensive, diabetes, atrial fibrillation, and coronary heart disease. The nomogram exhibited a high Hosmer–Lemeshow goodness-for-fit and good calibration (*P* > 0.05). The analysis, including the area under the receiver operating characteristic curve, the net reclassification index, the integrated discrimination improvement, and clinical use, showed that our prediction model was more accurate than the Framingham risk model in predicting stroke risk in RA patients. In conclusion, the nomogram can be used for individualized preoperative prediction of stroke risk in RA patients.

## INTRODUCTION

The morbidity rate of rheumatoid arthritis (RA), a leading cause of work disability, ranges from 0.32% to 0.36% in China. The highest morbidity of 0.5% has been reported in northeast China [1]. RA lesions are categorized into two major types—synovitis and pannus [2]. Synovitis refers to the inflammation of the synovial membrane lining a joint, whereas pannus involves the formation of irreversible pathological lesions in extra-articular joints and synovial tissue proliferation that can worsen the prognosis of RA. In addition, pannus RA increases the risk of cardiovascular disease (CVD) and other diseases such as stroke.

Elevated inflammatory levels and lipid abnormalities in RA patients are independent risk factors for atherosclerosis, stroke, and other CVD [3–6]. In addition, the risk of stroke among RA patients is associated with elevated levels of erythrocyte sedimentation rate (ESR), high-density lipoprotein (HDL) cholesterol [7], total cholesterol (TC), triglycerides (TG), anti-cyclic citrullinated peptide (anti-CCP) antibodies [8], low-density lipoprotein (LDL) cholesterol, and C-reactive protein (CRP) [8–11]. Although these quantitative descriptors are routinely detected in RA patients clinically and reported in electronic medical records (EMRs), clinicians lack the ability to interpret them and often ignore their clinical significance. Because most chronic diseases are a cumulative effect of several weak risk factors, statistically combining their effects could help to more robustly predict the risk and change the clinical management and decision-making process. Several risk prediction tools are increasingly being used in clinical medicine [12–14], such as the Framingham risk score (FRS) [15, 16], to determine clinical guidelines. The FRS tool for predicting cardiovascular events includes risk factors such as age, systolic blood pressure (SBP), diabetes mellitus, cigarette smoking, prior CVD, atrial fibrillation (AF), left ventricular hypertrophy (LVH), and the use of hypotensive medications. It is widely applicable (with calibration) to culturally diverse populations in Europe, the Mediterranean region, and Asia [17]. Similar effective coronary heart disease (CHD) risk prediction algorithms have been developed by other investigators worldwide [18–20].

We have previously reported that elevated ESR, LDL levels, and CRP levels ≥230 mg/L were independent risk factors for RA patients in developing stroke [21]. In addition, we developed and validated a nomogram to predict CHD in RA patients in northern China [22]. Numerous studies have reported a higher risk of stroke in RA patients compared with the general population. Here, we developed and validated a nomogram incorporating serum lipids and inflammatory markers for individual risk prediction of stroke in RA patients and compared its diagnostic and prognostic ability with that of FRS.

## RESULTS

### Baseline demographics and clinical characteristics

The study included 218 RA with stroke patients and 1,136 RA patients in the primary cohort. The validation cohort comprised 95 RA with stroke patients and 486 RA patients. The clinical characteristics of patients are listed in Table 1. The baseline clinical data were similar between primary and validation cohorts. As shown in Table 2, univariate LR analysis of RA patients developing stroke indicated that the stratification of sex, age, SBP, CRP, ESR, TC, LDL and the history of hypertension, diabetes, AF, CVD, and CHD, were significantly different between RA with stroke and RA groups (*P* < 0.05) in the primary cohort.

**Table 1:** Participants’ characteristics in primary and validation cohorts.

**Variables**	**Cohort No. (%)**		***χ²***	***P*-value**
	**Primary cohort (1,354)**	**Validation cohort (581)**		
RA with stroke	218 (16.10)	95 (16.35)	0.019	0.891
Sex, female	1021 (75.41)	428 (73.67)	0.655	0.419
Age, year				
18–65	757 (55.91)	308 (53.01)	1.386	0.500
66–79	426 (31.46)	194 (33.39)		
≥80	171 (12.63)	79 (13.60)		
SBP, mm Hg				
<120	422 (31.17)	194 (33.39)	7.511	0.111
120–139	592 (43.72)	240 (41.31)		
140–159	287 (21.20)	114 (19.62)		
160–179	46 (3.40)	24 (4.13)		
≥180	7 (0.52)	9 (1.55)		
Smoking	176 (13.00)	84 (14.46)	0.744	0.388
Diabetes	190 (14.03)	97 (16.70)	2.282	0.131
CHD	208 (15.36)	104 (17.90)	1.937	0.164
AF	40 (2.95)	17 (2.93)	0.001	0.973
LVH^*^	1 (0.07)	1 (0.17)	-	0.510
CVD	436 (32.20)	198 (34.08)	0.651	0.420
Hypertension	240 (17.73)	153 (26.33)	18.615	<0.001
Bio-med	22 (1.62)	12 (2.07)	0.457	0.499
CCP^+^	827 (61.08)	337 (58.00)	1.604	0.205
RF^+^	908 (67.06)	381 (65.58)	0.403	0.526
CRP, mg/L				
<10	319 (23.56)	157 (27.02)	3.769	0.152
≥9.06nd<64.32	670 (49.48)	287 (49.40)		
≥64.32	365 (26.96)	137 (23.58)		
ESR, mm/H				
<29	351 (25.92)	137 (23.58)	2.368	0.306
≥29nd<84.80	662 (48.89)	280 (48.19)		
≥84.8	341 (25.18)	164 (28.23)		
C3, g/L				
<0.95	345 (25.48)	155 (26.68)	0.890	0.641
≥0.95nd<1.34	673 (49.7)	293 (50.43)		
≥1.34	336 (24.82)	133 (22.89)		
C4, g/L				
<0.18	378 (27.92)	146 (25.13)	5.450	0.066
≥0.18nd<0.28	656 (48.45)	315 (54.22)		
≥0.28	320 (23.63)	120 (20.65)		
FBG, mmol/L				
<4.84	336 (24.82)	154 (26.51)	2.052	0.358
≥4.84nd<6.33	670 (49.48)	295 (50.77)		
≥6.33	348 (25.70)	132 (22.72)		
TC, mmol/L				
<5.2	1131 (83.53)	479 (82.44)	6.259	0.044
≥5.2nd<66.2	163 (12.04)	61 (10.50)		
≥6.2	60 (4.43)	41 (7.06)		
LDL, mmol/L				
<3.4	1106 (81.68)	463 (79.69)	6.278	0.043
≥3.4nd<4.1	182 (13.44)	73 (12.56)		
≥4.1	66 (4.87)	45 (7.75)		
HDL, mmol/L				
≥1.55	125 (9.23)	46 (7.92)	4.119	0.128
≥1.04nd<1.55	570 (42.10)	273 (46.99)		
<1.04	659 (48.67)	262 (45.09)		
TG, mmol/L				
<1.7	1,113 (82.20)	460 (79.17)	2.697	0.260
≥1.7nd<2.3	139 (10.27)	73 (12.56)		
≥2.3	102 (7.53)	48 (8.26)		

Data are represented as numbers and proportions. Statistics were calculated using the chi-square test. ^*^Statistics were calculated by Fisher’s exact test. Abbreviations: RA: rheumatoid arthritis; SBP: systolic blood pressure; CHD: coronary heart disease; AF: atrial fibrillation; LVH: left ventricular hypertrophy; CVD: cardiovascular disease; Bio-med: biologic disease-modifying anti-rheumatic drugs; CCP^+^: positive anti-cyclic citrullinated peptide antibody; RF^+^: positive rheumatoid factor; CRP: C-reactive protein; ESR: erythrocyte sedimentation rate; C3: complement 3; C4: complement 4; FBG: fasting blood glucose; TC: total cholesterol; LDL: low-density lipoprotein; HDL: high-density lipoprotein; TG: triglycerides.

**Table 2:** Univariate logistic regression analysis of RA patients developing stroke in the primary cohort.

**Variables**	**RA (1,136)**	**RA with stroke (218)**	**OR (95% CI)**	***P*-value**
Sex, female vs. male	873 (76.85)	148 (67.89)	0.64 (0.46–0.88)	0.005
Age, year				<0.001
66–79 vs. 18–65	336 (29.58)	90 (41.28)	2.90 (2.05–4.10)	
≥80 vs. 18–65	107 (9.42)	64 (29.36)	6.48 (4.33–9.68)	
SBP, mmHg				<0.001
120–139 vs.<120	503 (44.28)	89 (40.83)	1.45 (0.99–2.12)	
140–159 vs.<120	224 (19.72)	63 (28.90)	2.30 (1.52–3.48)	
160–179 vs.<120	29 (2.55)	17 (7.80)	4.79 (2.45–9.39)	
≥180 vs.<120	4 (0.35)	3 (1.38)	6.13 (1.33–28.25)	
Smoking	145 (12.76)	31 (14.22)	1.13 (0.75–1.72)	0.558
Diabetes	142 (12.50)	48 (22.02)	1.98 (1.37–2.85)	<0.001
CVD^*^	218 (19.19)	218 (100)	–	<0.001
CHD	130 (11.44)	78 (35.78)	4.31 (3.09–6.01)	<0.001
AF	22 (1.94)	18 (8.26)	4.56 (2.40–8.65)	<0.001
LVH^*^	0 (0)	1 (0.46)	-	0.161
Hypertension	164 (14.44)	76 (34.86)	3.17 (2.29–4.39)	<0.001
Bio-med	21 (1.85)	1 (0.46)	0.25 (0.03–1.83)	0.236
CCP^+^	698 (61.44)	129 (59.17)	0.91 (0.68–1.22)	0.529
RF^+^	766 (67.43)	142 (65.14)	0.90 (0.67–1.24)	0.510
CRP, mg/L				0.007
≥9.06 and <64.32 vs. <10	570 (50.18)	100 (45.87)	1.19 (0.81, 1.76)	
≥64.32 vs. <10	288 (25.35)	77 (35.32)	1.81 (1.20–2.74)	
ESR, mm/H				0.037
≥29 and <84.80 vs. <29	553 (48.68)	109 (50)	0.58 (0.38–0.88)	
≥84.8 vs. <29	275 (24.21)	66 (30.28)	0.82 (0.59–1.15)	
C3, g/L				0.516
≥0.95 and <1.34 vs. <0.95	567 (49.91)	106 (48.62)	0.85 (0.61–1.20)	
≥1.34 vs. <0.95	286 (25.18)	50 (22.94)	0.80 (0.53–1.20)	
C4, g/L				0.786
≥0.18 and <0.28 vs. <0.18	550 (48.42)	106 (48.62)	0.95 (0.67–1.33)	
≥0.28 vs. <0.18	272 (23.94)	48 (22.02)	0.87 (0.58–1.30)	
FBG, mmol/L				0.652
≥4.84 and <6.33 vs. <4.84	556 (48.94)	114 (52.29)	1.12 (0.78–1.60)	
≥6.33 vs. <4.84	296 (26.06)	52 (23.85)	0.96 (0.63–1.46)	
TC, mmol/L				0.038
≥5.2 and <66.2 vs. <5.2	143 (12.59)	20 (9.17)	0.73 (0.45–1.20)	
≥6.2 vs. <5.2	44 (3.87)	16 (7.34)	1.90 (1.05–3.43)	
LDL, mmol/L				0.004
≥3.4 and<4.1 vs. <3.4	138 (12.15)	44 (20.18)	1.87 (1.28–2.73)	
≥4.1 vs. <3.4	53 (4.67)	13 (5.96)	1.44 (0.77–2.70)	
HDL, mmol/L				0.774
≥1.04 and <1.55 vs. ≥1.55	483 (42.52)	87 (39.91)	0.89 (0.53–1.50)	
<1.04 vs. ≥1.55	549 (48.33)	110 (50.46)	0.99 (0.60–1.66)	
TG, mmol/L				0.186
≥1.7 and <2.3 vs. <1.7	114 (10.04)	25 (11.47)	1.11 (0.70–1.77)	
≥2.3 vs. <1.7	92 (8.10)	10 (4.59)	0.55 (0.28–1.08)	

Data are represented as numbers and proportions. Statistics were conducted using univariate logistic regression. Abbreviations: RA: rheumatoid arthritis; OR (95% CI), odds ratio, 95% confidence interval; SBP: systolic blood pressure; CHD: coronary heart disease; AF: atrial fibrillation; LVH: left ventricular hypertrophy; CVD: cardiovascular disease; Bio-med: biologic disease-modifying anti-rheumatic drugs; CCP^+^: positive anti-cyclic citrullinated peptide antibody; RF^+^: positive rheumatoid factor; CRP: C-reactive protein; ESR: erythrocyte sedimentation rate; C3: complement 3; C4: complement 4; FBG: fasting blood glucose; TC: total cholesterol; LDL: low-density lipoprotein; HDL: high-density lipoprotein; TG: triglycerides.

### Development of an individualized prediction model

**Figure 1:**
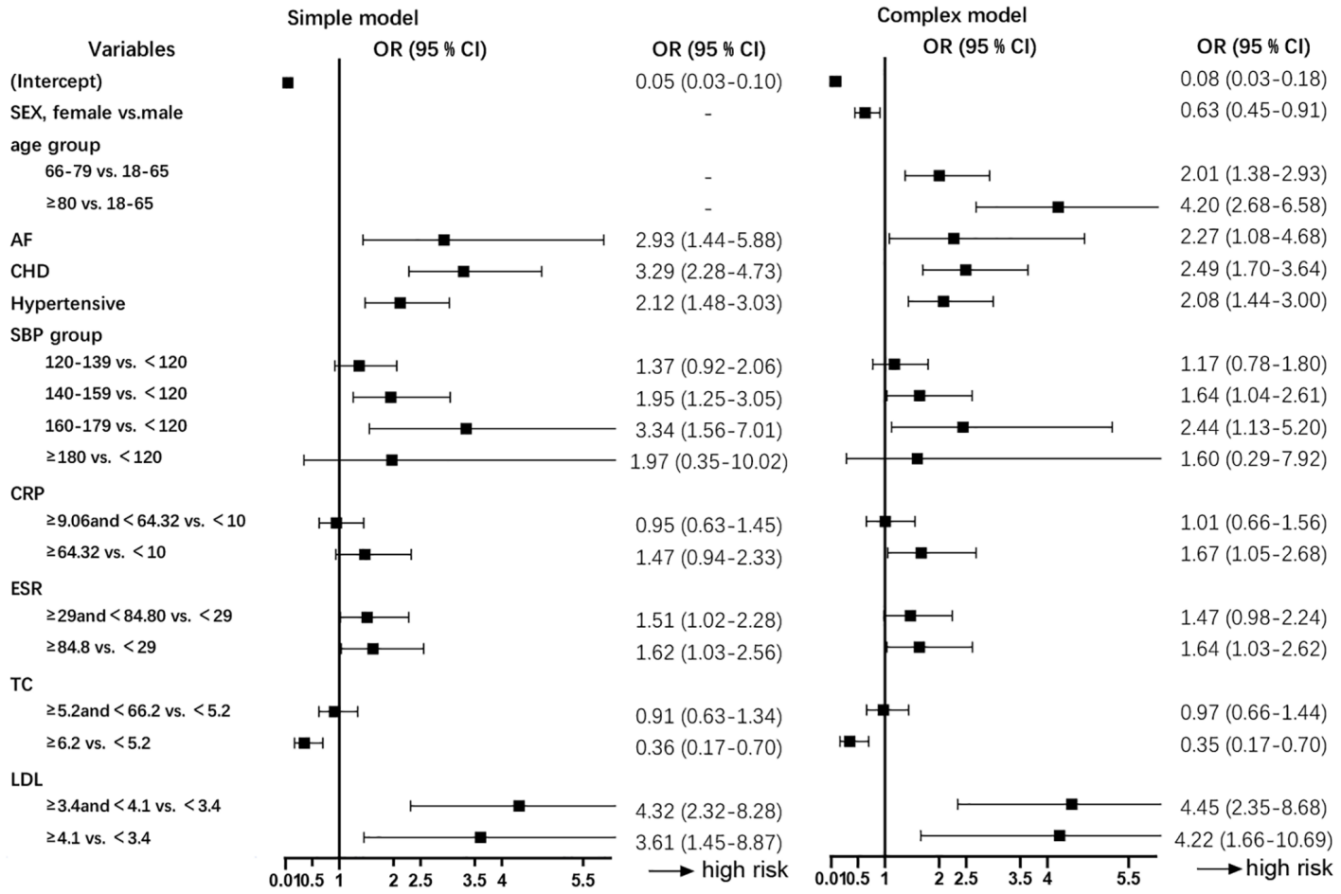
**Multivariate logistic regression analysis of data from RA patients developing stroke in the primary cohort (*N* = 1,354).** Abbreviations: SBP: systolic blood pressure; CHD: coronary heart disease; AF: atrial fibrillation; CRP: C-reactive protein; ESR: erythrocyte sedimentation rate; TC: total cholesterol; LDL: low-density lipoprotein; OR (95% CI): odds ratio, 95% confidence interval.

**Figure 2:**
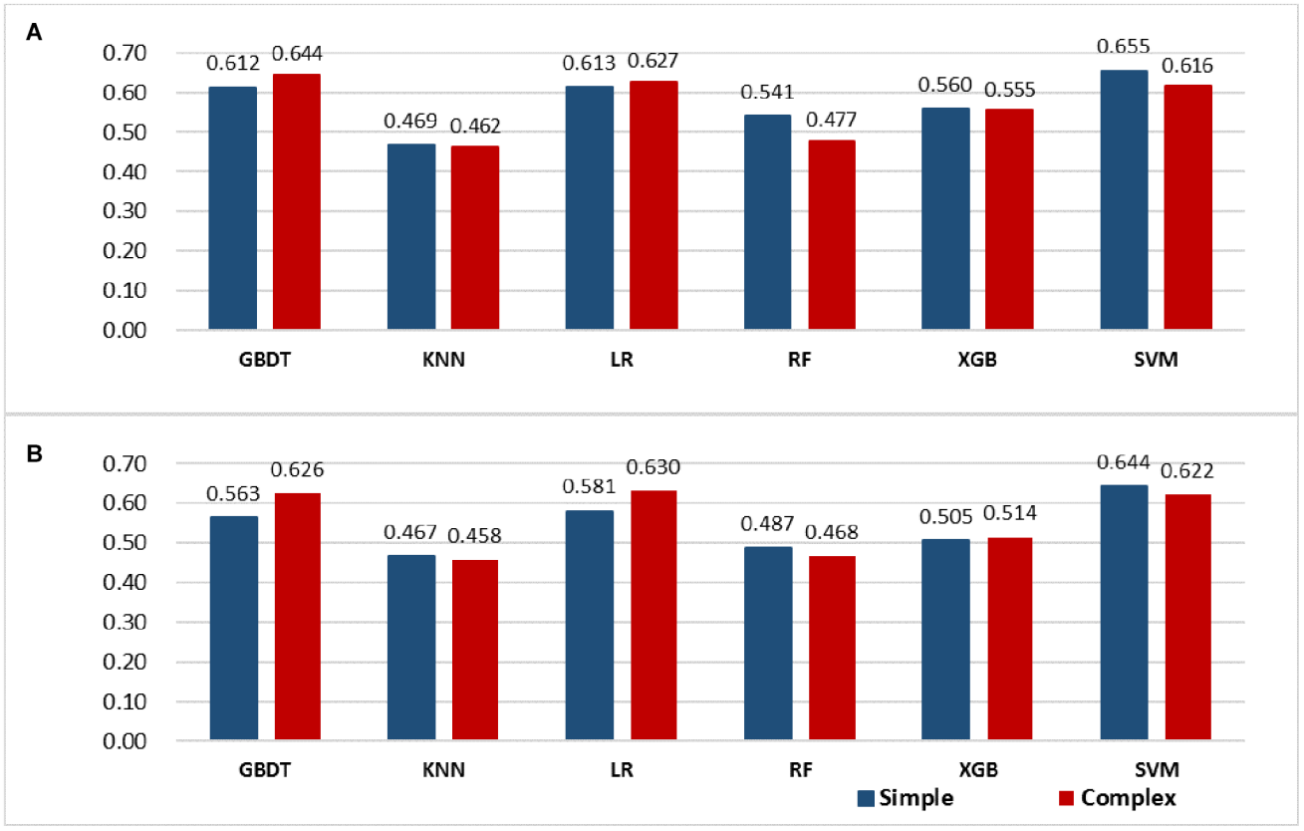
**Model evaluation (F1-score) results based on the number of features across six models.** (**A**) primary cohort, *N* = 1,354 patients; (**B**) validation cohort, *N* = 581 patients). Abbreviations: GBDT: gradient boosting decision tree; KNN: k-nearest neighbors; LR: logistic regression; RF: random forest; XGB: XGBoost; SVM: Support Vector Machine.

**Figure 3:**
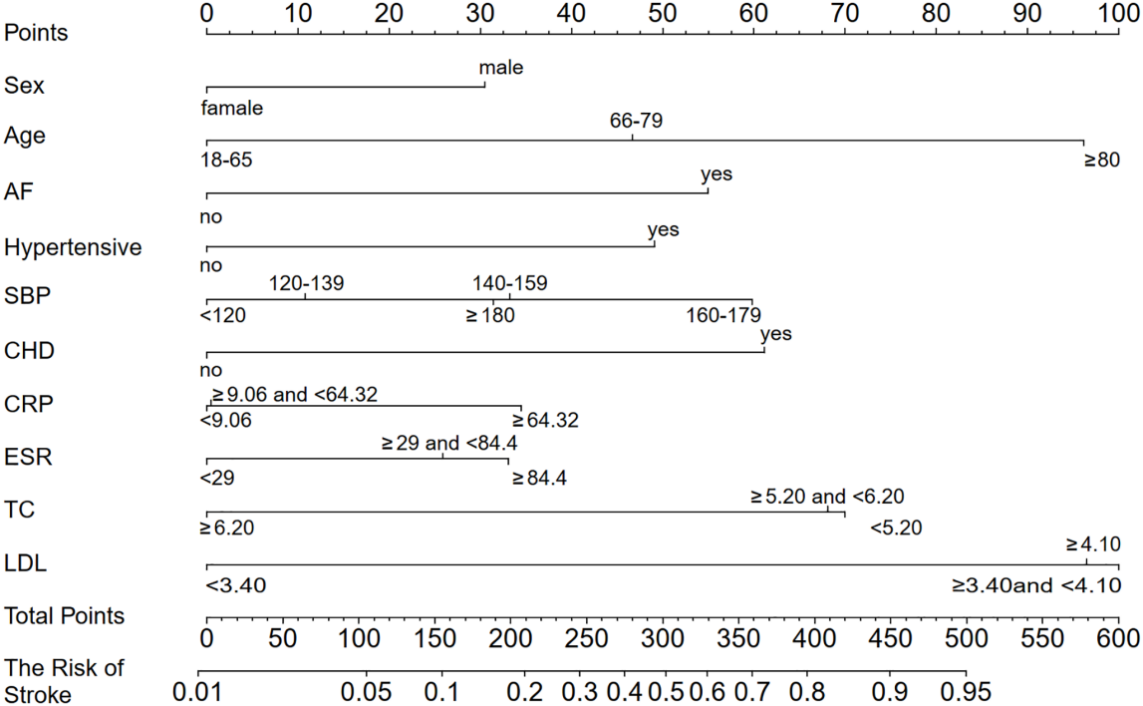
**A developed stroke nomogram in the primary cohort (*N* = 1,354).** Abbreviations: SBP: systolic blood pressure; CHD: coronary heart disease; AF: atrial fibrillation; CRP: C-reactive protein; ESR: erythrocyte sedimentation rate; TC: total cholesterol; LDL: low-density lipoprotein. For example, a 70-year-old (47 points), male (30 points) RA patient with an AF (55 points) and CHD (62 points) history of 60 mm/H ESR (27 points), and 5 mmol/L TC (65 points) arrived at a total point value of 286, with a probability of 46% of developing a stroke.

All variables in this analysis are listed in Table 2. These were carefully selected to ensure parsimony and practicality of the final model (noted in the Methods section). We finally identified the following 10 variables with the strongest association with stroke risk: sex, age, SBP, CRP, ESR, TC, LDL, and history of hypertension, diabetes, AF, and CHD. Based on the final complex model of multivariate analysis shown in Figure 1, several variables were independently associated with stroke among RA patients, such as sex (0.63 [0.45–0.91]), AF (2.27 [1.08–4.68]), CHD (2.49 [1.70–3.64]), hypertension (2.08 [1.44–3.00]), SBP stratification (for 140–159 vs.<120, 1.64 [1.04–2.61]; for 160–179 vs. <120, 2.44 [1.13–5.20]), CRP stratification (for ≥64.32 vs. <9.06, 1.67 [1.05–2.68]), ESR stratification (for ≥84.8 vs. <29, 1.64 [1.03–2.62]), TC stratification (for ≥6.2 vs.<5.2, 0.35 [0.17–0.70], LDL stratification (for 3.4–4.1 vs.<3.4, 4.45 [2.35–8.68]; for ≥4.1 vs.<3.4, 4.22 [1.66–10.69]). The final simple model of multivariate analysis is shown in Figure 1. A comprehensive analysis of vital indicators of machine learning models, shown in Figure 2 and Supplementary Table 1, reveals that the effect of the LR model on nomogram was better compared with other machine learning models. In addition, the LR algorithm effectively predicted the current data, simultaneously indicating the better efficiency of the complex model than the simple model. Finally, the complex model was used to create the nomogram by incorporating the above independent predictors (Figure 3). The nomogram revealed the score of influencing factors, the personal total cumulative score, and the predicted risk value of the individual outcome event for RA patients.

### Assessing the performance and internal validation of stroke nomogram

**Figure 4:**
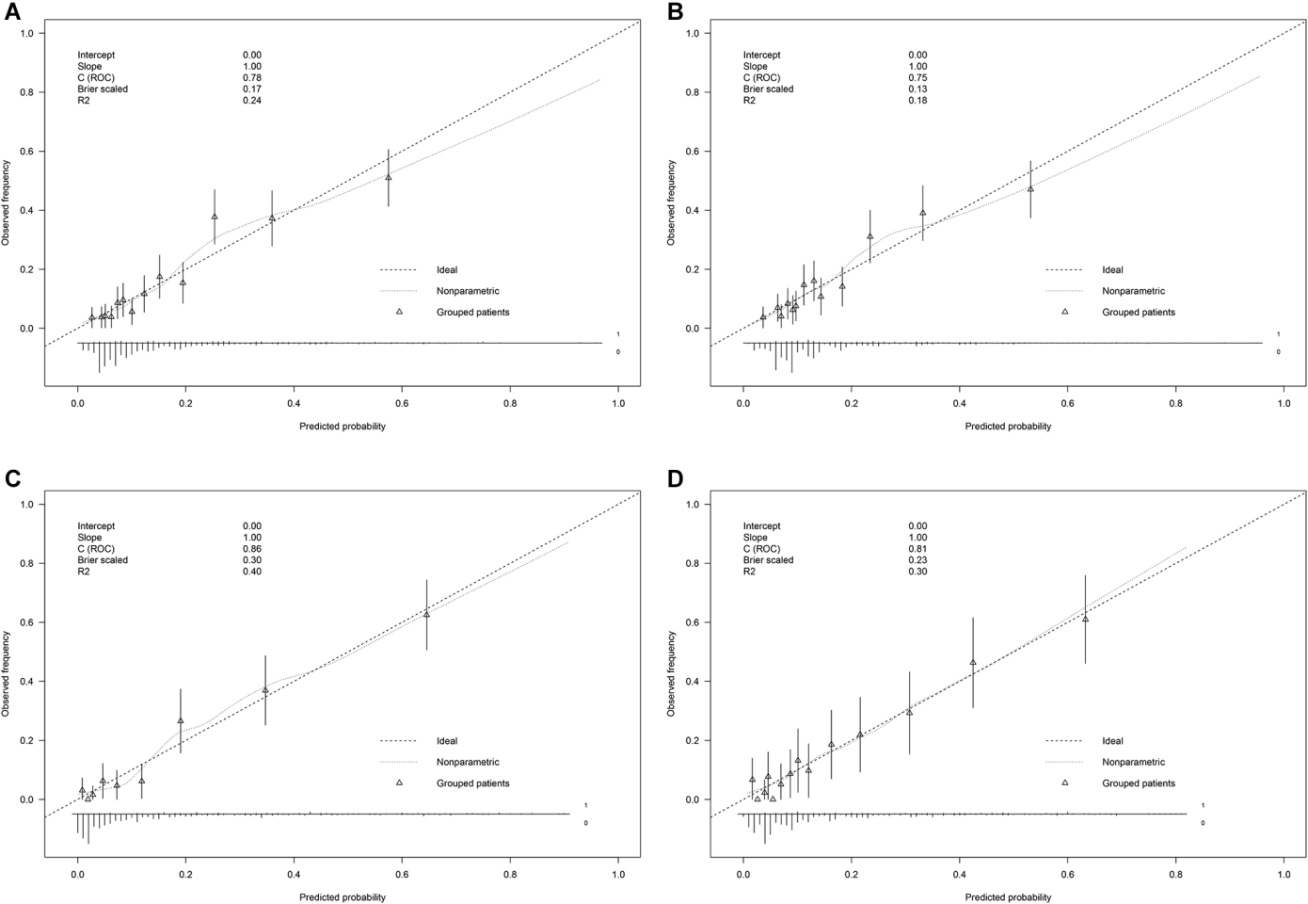
Calibration curves of (**A**) complex model in the primary cohort (*N* = 1,354), (**B**) simple model in the primary cohort (*N* = 1,354), (**C**) complex model in the validation cohort (*N* = 581), and (**D**) simple model in the validation cohort (*N* = 581). Calibration curves depicted the calibration of each model in an agreement between the predicted risks of stroke and observed outcomes of stroke. The y-axis represents the actual stroke. The x-axis represents the predicted stroke risk. The diagonal gray line represents the perfect prediction by an ideal model. The dotted line represents the performance of the nonparametric nomogram, of which a closer fit to the diagonal gray line represents a better prediction.

Figure 4 shows a good agreement between the predicted risk and observed outcomes in the primary and validation cohorts (slope = 1, intercept = 0 with simple and complex models). Further, we comprehensively assessed and compared the performance of developed models with the Framingham risk model (Table 3). The Hosmer–Lemeshow test showed no substantial deviation from perfect fit in simple versus complex model in the primary cohort (*P* = 0.385 vs. 0.097), revealing a good agreement in the probability of stroke between the predicted risk and observed outcomes in the primary cohort. The area under the receiver operating characteristic curve (AUC), used to measure the discrimination performance of models, showed that the complex model (AUC, 95% CI: 0.784 [0.750–0.818], *P* < 0.001) had a better diagnostic ability than the simple model (AUC, 95% CI: 0.747 [0.711–0.784], *P* < 0.001), *P* = 0.0016 and was different compared with the Framingham risk model (AUC, 95% CI: 0.808 [0.778–0.893], *P* < 0.001), *P* = 0.0631 in predicting the development of stroke in RA patients. In addition, the net reclassification indexes (NRI) and integrated discrimination indexes (IDI) were calculated based on comparing with the Framingham risk model, both simple and complex models correctly reclassified the subjects in predicting the stroke risk (NRI: 11.59 [2.90, 20.29], 20.30 [12.54, 28.05]. IDI:1.71 [–0.77, 4.18], 5.65 [3.41, 7.88] separately for simple and complex models vs. the Framingham risk model).

**Table 3:** Performance and internal validation of stroke nomogram.

	**Framingham risk model**	**Simple model**	**Complex model**
Hosmer–Lemeshow test			
*χ²*	NA	8.517	13.456
*P*	NA	0.385	0.097
AUC (95% CI)	0.808 (0.778, 0.839)^*&^	0.747 (0.711, 0.784)^*#^	0.784 (0.750, 0.818)^#&^
NRI (95% CI)	ref.	11.59 (2.90, 20.29)	20.30 (12.54, 28.05)
IDI (95% CI)	ref.	1.71 (−0.77, 4.18)	5.65 (3.41, 7.88)

^*^P = 0.0013, significant associations between Framingham risk and simple models; ^#^*P* = 0.0016, significant associations between complex and simple models; ^&^*P* = 0.0631, significant associations between complex and Framingham risk models. Abbreviations: AUC: area under the receiver operating characteristic curve; NRI: net reclassification index; IDI: integrated discrimination improvement; NA: not available; ref.: reference level.

### Clinical use of risk nomogram

The decision curve analysis (DCA) of the risk nomogram in the primary cohort (Supplementary Figure 2) indicated that when the risk threshold of stroke in RA patients was about 15%, intervention treatment was beneficial compared to either treat-all-patients or treat-none scheme. When the threshold value ranged from 15% to 55%, the net benefit was comparable based on the risk nomogram, suggesting that the performance of the complex model (blue line) was higher than that of the simple model (red line) in predicting the risk of stroke in RA patients.

## DISCUSSION

We used the EMR data from hospitalized patients in northern China to develop and validate a nomogram for predicting stroke risk in RA patients. The risk nomogram incorporated several factors, including sex, age, SBP, CRP, ESR, TC, LDL, and the history of hypertension, AF, and CHD.

Prediction models use multiple predictors to estimate the absolute probability or risk of disease outcomes in an individual within a specific time interval [23, 24]. Recently, certain study groups have used prediction models to create multi-markers for clinical decision-making. For example, Huang et al. reported a radiomics nomogram that incorporated the radiomics signature, computed tomography (CT)-reported lymph node status, and clinical risk factors to preoperatively predict lymph node metastasis in patients with colorectal cancer [13]. The FRS to predict stroke risk was calculated using patients’ 10-year data by Cox proportional hazards regression model [25], whereas we assessed and used real-world data. We evaluated six machine learning models and observed that the LR algorithm performed well, with better generalization, high accuracy and precision, good recall, and reduced balance error. Similar to multivariable analyses used in recent studies that incorporated individual markers into marker panels, our model connected multiple individual features by incorporating serum lipids and inflammatory markers. Furthermore, our model demonstrated adequate discrimination in a primary cohort, which was subsequently improved in the validation cohort. Our prediction model showed good calibration, with a high Hosmer–Lemeshow goodness-of-fit. Although the AUC analysis did not reveal a difference between complex and Framingham risk models, it showed that 20.30% [12.54, 28.05] of patients were correctly reclassified by the complex model than the Framingham risk model with 5.65% [3.41, 7.88] of IDI. DCA assessment is performed to evaluate model effectiveness and which alternative models should be used [26]. We used DCA to address the heterogeneity across different institutions in clinical data collection and subsequently to select the best model. When the risk threshold of stroke in RA patients was >15%, the net benefit of complex and simple models was superior to either treat-all-patients or treat-none scheme, being best for the complex model when the threshold probability was 15% to 55%. Altogether, these results showed that the easy-to-use nomogram effectively predicted the risk of stroke among RA patients, with strong clinical value for decision making for RA patients.

Several studies have reported that numerous demographic and clinical characteristics, including increased lipid metabolism levels, high inflammatory levels, and other traditional CVD risk factors, affect the risk of RA patients developing stroke [27–29]. However, these studies were conducted in Caucasians, lacking data from Asians. In addition, their results were inconsistent. For instance, Zhang et al. [27] hypothesized that RA-related systemic inflammation determined cardiovascular risk and a complex relationship between LDL and cardiovascular risk. Similarly, certain studies suggested the “lipid paradox” of LDL [7, 30], and others [9, 31] suggested that TC, LDL, and TG levels were specifically used to predict the risk of stroke in RA than in the general population. Our results emphasized the effects of serum TC and LDL levels in predicting the development of stroke among RA patients, simultaneously considering the traditional risk factors (hypertension, AF, and CHD history) reported in previous studies on FRS [16, 25, 32]. For this particular RA population, our findings underscored the contribution of systemic inflammation to stroke development in RA patients, with CRP and ESR inflammatory factors as independent risk factors for stroke in RA patients, also confirmed previously [4, 33]. Because multiple factors contribute to stroke development in RA patients, a cumulative effect of several weak risk factors could more reliably predict the risk than a single risk factor. Thus, we developed a nomogram that incorporated several independent risk predictors.

Our study had several limitations. First, the data used were obtained from a single center. The results need to be externally validated by other centers as well. Therefore, we computed the C-index for the prediction nomogram via bootstrapping validation and assessing the NRI of a bootstrap resample with 1,000 individuals for multiple validations. Second, our study was completely based on clinical data; additional biomarkers could further improve the diagnostic accuracy of the model. Third, with recent studies reporting an association between RA and stroke, it is unclear if a model based on traditional risk factors, serum lipids, and inflammatory markers could effectively predict outcomes. Well-designed randomized clinical trials or cohorts are required to further compare the predictive ability of our model with FRS for stroke in RA patients. Clinical prediction models can guide the professionals in decision-making, thereby improving patient outcomes and cost-effectiveness of care. In addition, prediction models objectively estimate the risks for both individuals (patients) and healthcare providers, thus assisting in subjective interpretations and intuitions, and building guidelines [34, 35].

In conclusion, we developed an effective nomogram that incorporates the traditional risk factors, serum lipids, and inflammatory markers to predict stroke in RA patients. We believe our study will provide a theoretical basis for improving the prognosis of RA patients and preventing the onset of stroke.

## MATERIALS AND METHODS

### Patient selection

A total of 8,389 RA patients, with 9.04% (758) prevalence of stroke, were selected from the inpatient Department of Rheumatology and Immunology of the First Affiliated Hospital of China Medical University from January 2011 to December 2018. According to the inclusion and exclusion criteria, 313 RA with stroke patients and 1,827 RA patients were selected (Supplementary Figure 1), aged 18 years or older. EMRs were classified and coded using the International Classification of Diseases Tenth Revision (ICD-10) of the Beijing clinical version (RA: M05.x~06.x; stroke [ischemic and hemorrhagic]: I60 I60.1–I60.0 I61 I61.0–I61.9 I69.0 I69.1 I63 I63.0–I63.9 I69.3). The study conformed to the principles outlined in the Declaration of Helsinki and was approved by the ethics committee of the Medical Science Research Institute of the First Affiliated Hospital, China Medical University (approval number: AF-SOP-07-1.0-01). All patients provided written informed consent for the use of their data.

The study criteria included the American College of Rheumatology (ACR) 1987/2010 [36] for RA and the cardiac vascular disease (CVD) criteria adopted at the Fourth Academic Conference by the Chinese Neuroscience Society in 1995 [37] for stroke. In the RA with stroke cohort, patients who met the following inclusion criteria were enrolled: 1) patients who conformed to the above stroke and RA diagnostic criteria; 2) if the timing of stroke was later than that of RA in the EMR, we believed that the patient had developed stroke after being diagnosed with RA; 3) patients had undergone laboratory tests (serum inflammatory, antibody, complement, lipid-assays) at least once when visited the hospital the first time; and 4) patients were older than 18 years. The RA cohort (without stroke) included patients who met the following inclusion criteria: 1) those who conformed to the above RA diagnostic criteria; 2) patients had undergone laboratory tests (serum inflammatory, antibody, complement, lipid assays) at least once when they first visited the hospital; and 3) patients were older than 18 years. The following exclusion criteria were applied to both cohorts: 1) patients who still suffered from other connective tissue diseases, including systemic lupus erythematosus, scleroderma, dry syndrome, and vasculitis; and 2) RA patients with coexisting ankylosing spondylitis and gout arthritis. Finally, we randomly selected 70% of the RA with stroke and RA patients as the primary cohort; the remaining patients comprised the validation cohort [38].

### Data collection

All data were filtered from EMR, and primarily included personal information, such as age, gender, height, and weight; metabolic indices (serum TC, TG, LDL, and HDL); fasting blood glucose (FBG); serologic profiles including CRP, ESR, rheumatoid factor (RF), complement3 (C3), complement4 (C4), and anti-cyclic citrullinated peptide (anti-CCP) antibodies, and CHD, AF, LVH, and CVD history records. In addition, we included the medication history, i.e., hypertension and biologic disease-modifying anti-rheumatic drugs (Bio-med). All laboratory tests were performed using overnight fasting venous blood samples and conducted using clinical standard operating procedures (SOP) for inspection items. In addition, when results of multiple laboratory tests at different time points were summarized, the first laboratory test results were selected at first admission for RA with stroke cohort and the RA cohort.

### Statistical analysis

All reported significant levels were set at 0.05 with a two-sided test. The categorical data are expressed as percentages by cohort. Certain continuous predictors (i.e., age, SBP, and CRP) were categorized using consensus approaches, guidelines, or previously published studies. In addition, the absence of certain features in clinical medical records was inevitable and accounted for less than 20%; we used multiple imputations to account for the missing data in SPSS 23.0. Chi-square test or Fisher’s exact test was used to compare the differences in participants’ characteristics between primary and validation cohorts. The univariable association between RA with stroke group and RA group of the primary cohort was assessed using univariate LR analysis. Based on this and clinical importance, scientific knowledge, and predictors identified previously [39], we developed and validated the model for predicting the risk of developing stroke in RA patients. The study report was built according Transparent Reporting of a multivariable prediction model for Individual Prognosis Or Diagnosis (TRIPOD) checklist for development and validation of the prediction model (https://www.equator-network.org/reporting-guidelines/tripod-statement/), shown as Supplementary Figure 3.

### Developing the model between the RA with stroke group and RA group in the primary cohort

The LR algorithm was used to develop an unadjusted simple model and a complex model adjusted by sex and age. We considered the disparity between male and female morbidity in RA patients and the aging of stroke patients. Further, we built the risk nomogram using multivariable LR analysis to provide the clinicians with a quantitative tool to predict the individual probability of stroke. All analyses were conducted using the R software version 3.6.2 (packages primarily including rms, Hmisc, dca. R packages; http://www.rproject.org).

### Comparing multiple machine learning algorithms in primary and validation cohorts

Machine learning algorithms were used based on scikit-learn, an open-source machine learning library, using Bayesian optimization to implement algorithm optimization, and cross-validation method (N-folds = 5) to complete algorithm evaluation during optimization. We used six different machine algorithms running for three 30-min sessions, including LR, Support Vector Machine (SVM), random forest (RF), XGBoost (XGB), gradient boosting decision tree (GBDT), and k-nearest neighbors (KNN), to compare algorithms and evaluate simple and complex models in primary and validation cohorts, respectively. The cohorts were compared by evaluation metrics as follows: 1) accuracy: the proportion of patients who were predicted as their actual status; 2) precision: the proportion of patients who actually had the disease and were predicted out of patients who were predicted as having the disease; 3) recall: the proportion of patients who were predicted as having disease out of all patients who actually had the disease, corresponding to sensitivity; 4) F1-score: the harmonic mean of precision and recall. F1 = 2Precision × recall/(precision + recall); and 5) balance error (ber): combining bias and variance to reflect the accuracy of the model.

### Comparing and validating the performance of developed models

The performance of the nomogram of the model was assessed by discrimination and calibration curves. Calibration curves, the accuracy of point estimates of the LR function, and the Hosmer–Lemeshow test were used to assess if the model calibrated perfectly. The discrimination of the nomogram was evaluated using C-index. The predictive accuracy for individual outcomes (discriminating ability) was equivalent to the AUC and was compared between the Framingham risk model and our prediction models. The models were internally validated using the validation cohort. The LR formula formed in the primary cohort was applied to all patients of the validation cohort. The NRI indicated the proportion of patients correctly reclassified by the new model compared with an existing or standard model, whereas IDI indicated the change in the difference in average predicted probabilities between RA with stroke and RA groups in the new and existing models [40]. Further, NRI and IDI between the Framingham risk model and our prediction models were assessed as having low risk (0–20%), medium risk (20%–59%), and high risk (60%–100%).

### Clinical use

DCA was assessed in the primary cohort to evaluate the clinical usefulness of the nomogram by quantifying the net benefits at different threshold probabilities. These results were used to identify the predictive models with the best discriminative abilities [41]. In addition, net benefit was defined as the proportion of true positives minus the proportion of false positives, weighted by the relative harm of false-positive and false-negative results [42]. Simple and complex models were used to predict risk stratification of 1,000 individuals using bootstrapping and assessing clinical impact with decision curves.

## Supplementary Materials

Supplementary Figures

Supplementary Table 1
