# Supplementary material for: Development and validation of a nomogram for predicting stroke risk in rheumatoid arthritis patients
Source: Aging (Albany NY). 2021 Jun 3;13(11):15061–77. doi: 10.18632/aging.203071 (PMC8221354; doi:10.18632/aging.203071)
Supplement: Supplementary Figures [file aging-13-203071-s001.pdf]

SUPPLEMENTARY FIGURES

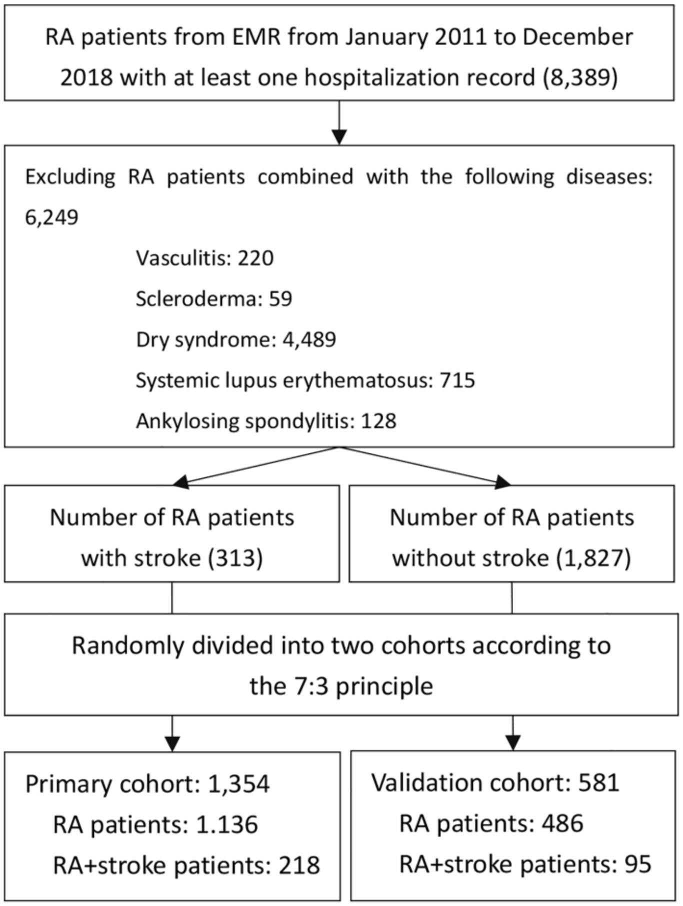

Supplementary Figure 1. Flow chart showing the selection process of study participants (*N* = 8,389). Abbreviations: RA: rheumatoid arthritis; EMR: electronic medical record.

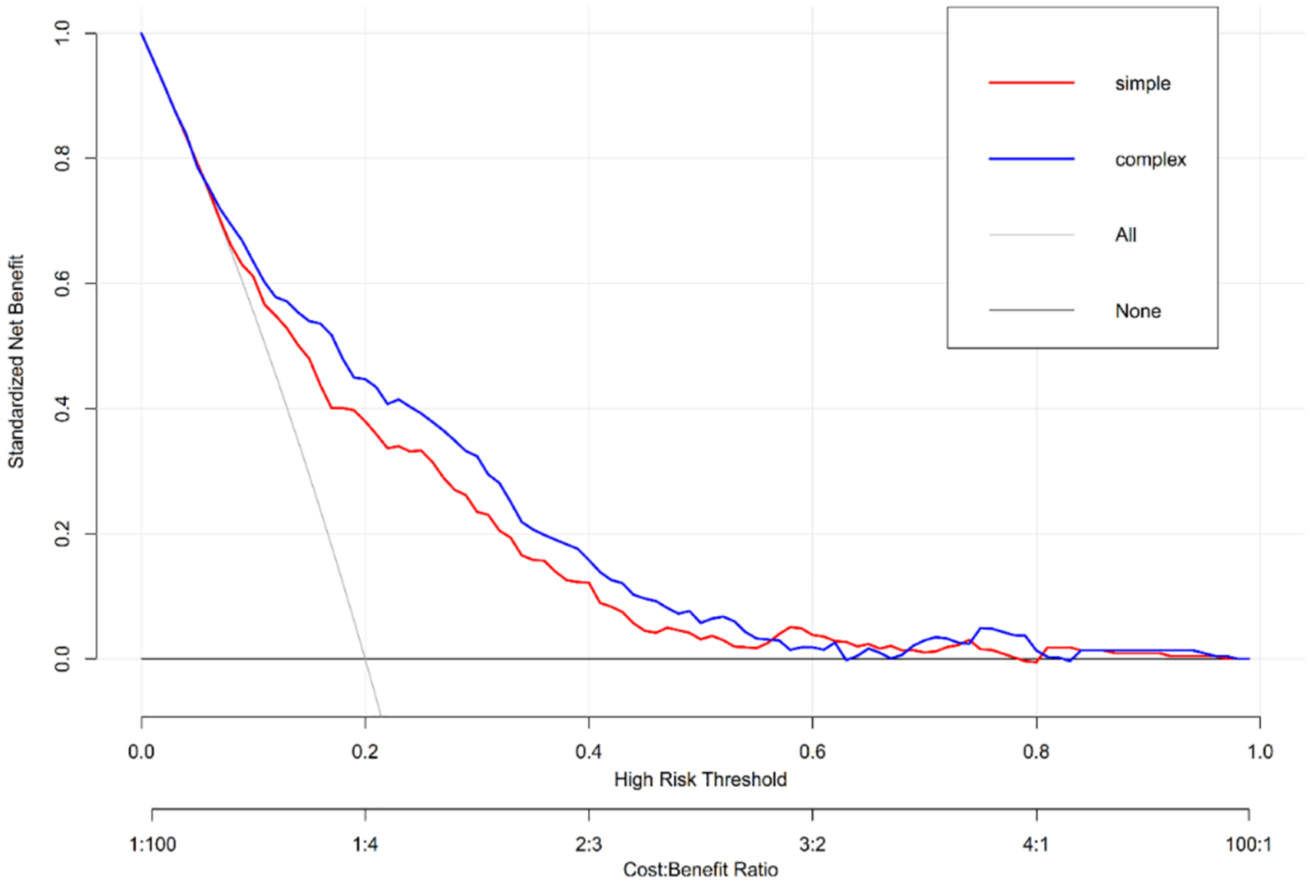

Supplementary Figure 2. Decision curve analysis for serum lipids, inflammatory markers, and serological status in rheumatoid arthritis and stroke patients in simple and complex models in the primary cohort (*N* = 1,354). The y-axis represents the net benefit, the x-axis represents the risk threshold of stroke in RA patients. The red line represents the nomogram of predictors in the simple model. The blue line represents the complex model with the addition of sex and age. The gray line represents the assumption that all patients had a stroke. The thin black line represents the assumption that no RA patient developed stroke. The net benefit was calculated by subtracting the proportion of all patients who were false positive from the proportion who were true positive, weighting by the relative harm of forgoing treatment compared with the negative consequences of unnecessary treatment.

Section/Topic	Item	Development or Validation?	Checklist Item
**Title and abstract**			
Title	1	D;V	Identify the study as developing and/or validating a multivariable prediction model, the target population, and the outcome to be predicted.
Abstract	2	D;V	Provide a summary of objectives, study design, setting, participants, sample size, predictors, outcome, statistical analysis, results, and conclusions.
**Introduction**			
Background and objectives	3a	D;V	Explain the medical context (including whether diagnostic or prognostic) and rationale for developing or validating the multivariable prediction model, including references to existing models.
	3b	D;V	Specify the objectives, including whether the study describes the development or validation of the model, or both.
**Methods**			
Source of data	4a	D;V	Describe the study design or source of data (e.g., randomized trial, cohort, or registry data), separately for the development and validation datasets, if applicable.
	4b	D;V	Specify the key study dates, including start of accrual; end of accrual; and, if applicable, end of follow-up.
Participants	5a	D;V	Specify key elements of the study setting (e.g., primary care, secondary care, general population) including number and location of centres.
	5b	D;V	Describe eligibility criteria for participants.
	5c	D;V	Give details of treatments received, if relevant.
Outcome	6a	D;V	Clearly define the outcome that is predicted by the prediction model, including how and when assessed.
	6b	D;V	Report any actions to blind assessment of the outcome to be predicted.
Predictors	7a	D;V	Clearly define all predictors used in developing the multivariable prediction model, including how and when they were measured.
	7b	D;V	Report any actions to blind assessment of predictors for the outcome and other predictors.
Sample size	8	D;V	Explain how the study size was arrived at.
Missing data	9	D;V	Describe how missing data were handled (e.g., complete-case analysis, single imputation, multiple imputation) with details of any imputation method.
Statistical analysis methods	10a	D	Describe how predictors were handled in the analyses.
	10b	D	Specify type of model, all model-building procedures (including any predictor selection), and method for internal validation.
	10c	V	For validation, describe how the predictions were calculated.
	10d	D;V	Specify all measures used to assess model performance and, if relevant, to compare multiple models.
	10e	V	Describe any model updating (e.g., recalibration) arising from the validation, if done.
Risk groups	11	D;V	Provide details on how risk groups were created, if done.
Development vs. validation	12	V	For validation, identify any differences from the development data in setting, eligibility criteria, outcome, and predictors.
**Results**			
Participants	13a	D;V	Describe the flow of participants through the study, including the number of participants with and without the outcome and, if applicable, a summary of the follow-up time. A diagram may be helpful.
	13b	D;V	Describe the characteristics of the participants (basic demographics, clinical features, available predictors), including the number of participants with missing data for predictors and outcome.
	13c	V	For validation, show a comparison with the development data of the distribution of important variables (demographics, predictors, and outcome).
Model development	14a	D	Specify the number of participants and outcome events in each analysis.
	14b	D	If done, report the unadjusted association between each candidate predictor and outcome.
Model specification	15a	D	Present the full prediction model to allow predictions for individuals (i.e., all regression coefficients, and model intercept or baseline survival at a given time point).
	15b	D	Explain how to use the prediction model.
Model performance	16	D;V	Report performance measures (with CIs) for the prediction model.
Model updating	17	V	If done, report the results from any model updating (i.e., model specification, model performance).
**Discussion**			
Limitations	18	D;V	Discuss any limitations of the study (such as nonrepresentative sample, few events per predictor, missing data).
Interpretation	19a	V	For validation, discuss the results with reference to performance in the development data, and any other validation data.
	19b	D;V	Give an overall interpretation of the results, considering objectives, limitations, results from similar studies, and other relevant evidence.
Implications	20	D;V	Discuss the potential clinical use of the model and implications for future research.
**Other information**			
Supplementary information	21	D;V	Provide information about the availability of supplementary resources, such as study protocol, Web calculator, and datasets.
Funding	22	D;V	Give the source of funding and the role of the funders for the present study.

* Items relevant only to the development of a prediction model are denoted by *D*, items relating solely to a validation of a prediction model are denoted by *V*, and items relating to both are denoted *D;V*. We recommend using the TRIPOD checklist in conjunction with the TRIPOD explanation and elaboration document.

Supplementary Figure 3. Transparent Reporting of a multivariable prediction model for Individual Prognosis Or Diagnosis (TRIPOD) checklist for development and validation of the prediction model. (https://www.equator-network.org/reporting-guidelines/tripod-statement/)
