# Supplementary material for: Development and validation of a nomogram for predicting stroke risk in rheumatoid arthritis patients
Source: Aging (Albany NY). 2021 Jun 3;13(11):15061–77. doi: 10.18632/aging.203071 (PMC8221354; doi:10.18632/aging.203071)
Supplement: Supplementary Table 1 [file aging-13-203071-s002.pdf]

SUPPLEMENTARY TABLE

Supplementary Table 1. Comparison of several machine learning models between the RA with stroke and RA groups.

	Training					Validation				
	**Accuracy**	**F1-score**	**Recall**	**Precision**	**Ber**	**Accuracy**	**F1-score**	**Recall**	**Precision**	**Ber**
**Simple**										
GBDT	0.835	0.612	0.611	0.789	0.389	0.815	0.563	0.581	0.690	0.419
KNN	0.840	0.469	0.506	0.616	0.494	0.836	0.467	0.505	0.595	0.495
LR	0.778	0.613	0.651	0.705	0.349	0.752	0.581	0.641	0.658	0.359
RF	0.851	0.541	0.547	0.847	0.453	0.834	0.487	0.515	0.640	0.485
XGB	0.854	0.560	0.557	0.863	0.443	0.837	0.505	0.523	0.630	0.477
SVM	0.763	0.655	0.711	0.641	0.289	0.738	0.644	0.716	0.636	0.284
**Complex**										
GBDT	0.842	0.644	0.636	0.778	0.364	0.826	0.626	0.629	0.718	0.371
KNN	0.840	0.462	0.503	0.620	0.497	0.836	0.458	0.501	0.468	0.499
LR	0.710	0.627	0.721	0.632	0.279	0.694	0.630	0.756	0.647	0.244
RF	0.842	0.477	0.510	0.580	0.490	0.838	0.468	0.506	0.505	0.494
XGB	0.854	0.555	0.557	0.885	0.443	0.842	0.514	0.531	0.629	0.469
SVM	0.773	0.616	0.678	0.599	0.322	0.765	0.622	0.703	0.608	0.297

Abbreviations: ber: balance error; LR: logistic regression; SVM: Support Vector Machine; RF: random forest; XGB: XGBoost; GBDT: gradient boosting decision tree; KNN: k-nearest neighbors.
